# Experimentation on Death from Chloroform

**Published:** 1877-04-20

**Authors:** Benjamin W. Richardson


### EXPERIMENTATION ON DEATH FROM CHLOROFORM.

The first series of experiments I remember to have made were commenced in the years 1850 and '51, and had reference to the mode and cause of death under chloroform. At the time named chloroform had been in use a little over two years for preventing the pain of surgical operations, and already nineteen deaths in man had occurred from it.

These calamities had produced very painful and anxious feelings amongst medical men, and my researches had for their intention the elucidation of many points of practical importance. The mode of procedure was to narcotize the animals, with varying degrees of rapidity, with varying percentages of chloroform vapor in the atmosphere, and during various atmospherical conditions; to note carefully the phenomena produced on the heart and on the respiration, and the duration of the four stages of narcotism. In some instances the animals—rabbits were usually subjected to experiment—were allowed to recover; in other instances the narcotism was continued to death. When the narcotism was made to be fatal the immediate cause of death was noted, and the body was left until the rigidity of death could be recorded. Then all the organs were carefully inspected, in order to see what was the condition of the lungs, the heart, the brain, the spinal cord.

The results obtained by these inquiries were of direct practical value. By them I showed in various lectures and papers the following major facts:

1. That the cause of the fatality from chloroform does not occur, as was at first supposed, from any particular mode of administration of the narcotic.

2. That chloroform will kill, in some instances, when the subject killed by it exhibits, previous to administration, no trace of disease or other sign by which the danger of death can be foretold.

3. That the condition of the air at the time of administration materially influences the action of the narcotic vapor. That the danger of administration is much less when the air is free of water vapor and the temperature is above 60°, but below 70° Fahr.

4. That there are four distinct modes of death from chloroform, and that when the phenomena of death from its application appear, they are infinitely more likely to pass into irrevocable death than from some other narcotics that may be used in lieu of chloroform.

5. That all the members of the group of narcotic vapors of the chlorine series, of which chloroform is the most prominent as a narcotic, are dangerous narcotics, and that chloroform ought to be replaced by some other agent equally practical in use and less fatal.

6. That so long as it continues to be used there will always be a certain distinct mortality arising from chloroform, and that no human skill in applying it can divest it of its dangers.

That knowledge of this kind respecting an agent which destroys one person out of every two thousand five hundred who inhale it was calculated to be useful, no reasonable mind, I think, can doubt. To me, who, many hundred times in my life, have had the solemn responsibility of administering chloroform to my fellow-men, it was of so much value that I should have felt it a crime if I had gone blindly on using so potent an instrument without obtaining such knowledge.—BENJAMIN W. RICHARDSON *in Nature.*

